# Using native human sera to assess the suitability of external quality assessment materials based on testosterone, progesterone, and 17β-estradiol measurements

**DOI:** 10.3389/fmolb.2026.1758376

**Published:** 2026-05-13

**Authors:** Laura Vierbaum, Patricia Kaiser, Thomas Keller, Stephan Weber, Ingo Schellenberg, Peter B. Luppa, Folker Wenzel, Mario Thevis

**Affiliations:** 1 INSTAND e.V., Society for Promoting Quality Assurance in Medical Laboratories, Duesseldorf, Germany; 2 Institute of Biochemistry/Center for Preventive Doping Research, German Sport University Cologne, Cologne, Germany; 3 ACOMED statistik, Leipzig, Germany; 4 Institute of Clinical Chemistry and Pathobiochemistry, University Hospital Rechts der Isar, Technische Universität München, Munich, Germany; 5 Faculty of Medical and Life Sciences, Furtwangen University, Villingen Schwenningen, Germany

**Keywords:** 17β-estradiol, commutability, comparative EQA studies, native external quality assessment (EQA) material, progesterone, steroid hormones, testosterone

## Abstract

**Introduction:**

The commonly processed nature of the control materials used in the external quality assessment (EQA) of medical laboratories requires that their suitability is evaluated against native patient samples to ensure valid evaluation of EQA results across several measurement procedures (MPs). The aim of this study was to examine the extent to which EQA analyses of a small number of native human samples in a regular EQA survey can help to assess the suitability of routinely distributed processed EQA materials (EQAMs), demonstrated by testosterone, progesterone, and 17β-estradiol measurements in human sera.

**Methods:**

Laboratory results for the native and processed EQAMs were comparatively analyzed using a mixed-effects model. This estimated the sample type-specific mean natural logarithm (ln) bias to the reference measurement value and mean differences in ln bias between the two sample types, each with a 95%-confidence interval. Applying quite strict commutability criteria defined in consideration of clinical guidelines enabled the identification of analyte/reagent manufacturer pairings exhibiting only minor differences in bias between sample types. For pairings showing differences in bias between the two sample types that exceeded the commutability criterion, efforts were made to differentiate between material- and MP-related contributions to the model outcomes – referred to as non-commutability and trueness bias, respectively.

**Results:**

Strongly scattered bias of the native EQAMs for some MPs revealed issues with measurement performances, such as inappropriate calibration or specificity. Consistent bias but distinct differences in bias between sample types indicated issues with commutability. The two-stage assessment revealed that the processed EQAMs were suitable for EQA purposes in most pairings. In two exceptions (testosterone/DG and progesterone/BE), the processed materials showed insufficient commutability. The limited number of EQAMs may result in inconclusive commutability assessments for individual analyte/MP pairings, four in this study, but allows for targeted follow-up with additional native EQAMs or comprehensive commutability studies.

**Conclusion:**

Compared to conventional commutability studies, these proposed EQA comparability studies that use comparative native EQAMs offer EQA providers a more efficient and resource-conserving approach to assess the suitability and commutability of EQAMs by leveraging existing infrastructures. Nevertheless, even this more pragmatic approach remains resource-intensive and is inherently restricted to specific questions and selected analytes.

## 1 Introduction

Continuous quality control of analytical performance in medical laboratories is essential to ensure reliable analyses and, ultimately, accurate diagnoses and effective therapies for patients. The results of external quality assessment (EQA) schemes provide information on the comparability of measurement values between laboratories and give a valuable indication of the status of standardization across different measurement procedures (MPs) (Ceriotti, 2014; Miller et al., 2011b; Vierbaum et al., 2024b; Wojtalewicz et al., 2023). To allow conclusive statements to be drawn from EQA programs on the quality and homogeneity of analytical testing in medical laboratories, it is essential to establish the suitability of the control samples (EN-ISO/IEC, 2023). EQA materials (EQAMs) typically consist of pooled, spiked, stabilized, and/or lyophilized patient specimens. This enables to be provided within an ethically acceptable framework sufficient sample quantities at clinically relevant concentrations and with an improved stability.

To ensure a valid EQA evaluation, however, the processing of the material should not introduce any substantial MP-specific deviations to the measurement results compared to those obtained for patient samples (Badrick et al., 2018; Delatour et al., 2016; Jennings et al., 2019; Miller et al., 2011a; Miller et al., 2006; Miller et al., 2018; Nilsson et al., 2018). Such effects may result from structural changes to the analyte, e.g., in case of proteins (Butreddy et al., 2021), interferences with the analytical method due to added substances, e.g., anticoagulants in hormone measurements (Kohek et al., 2002), or general matrix effects (Kim et al., 2019; Miller, 2003; Miller et al., 2011a). Commutability is particularly crucial for EQA evaluations if a reference measurement procedure (RMP) is available, because the accuracy of the laboratory results can then be evaluated using a reference measurement value (RMV) as the target value instead of an MP-specific consensus value (Ceriotti, 2014; Franzini and Ceriotti, 1998; Miller, 2003; Miller et al., 2011a).

Given the high production frequency of EQAMs, resource-intensive studies that assess the commutability of control materials in accordance with reported recommendations cannot be broadly applied. Therefore, EQA providers require more practical approaches (Vierbaum et al., 2024a). The aim of this study was to examine the potential of comparative EQA analyses by using limited number of native EQAMs and routinely distributed processed EQAMs–here exemplified by testosterone, progesterone, and 17β-estradiol–to assess the suitability of the processed materials. But even EQA studies with comparative native samples require considerable resources, making them only appropriate for specific questions and analytes. Previous EQA surveys conducted by INSTAND – Society for Promoting Quality in Medical Laboratories e.V. have revealed notable differences in immunoassay results depending on the reagent manufacturer for all three steroid hormones, as well as considerable deviations from the RMV for individual reagents (Vierbaum et al., 2024b). This study examined the extent to which the previously observed MP-specific differences were related to non-commutability bias, e.g., due to matrix effects, or to trueness bias depending on the MP performances (Miller et al., 2008).

## 2 Materials and methods

### 2.1 Processed EQAMs

The EQAMs for the measurement of testosterone, progesterone and 17β-estradiol were provided by INSTAND and were prepared from pooled human sera. To achieve specific concentrations, the sera were spiked with the respective steroid hormones and stabilized using proprietary additives. After equilibration, the material was preserved with 0.02% sodium azide and aliquoted to 2 mL vials. The samples were stored at 2 °C–8 °C in liquid form until they could be dispatched to the EQA participants. The homogeneity and stability of the EQAMs were confirmed to be in accordance with DIN EN ISO/IEC 17043:2010.

### 2.2 Pooled native EQAMs

The native pooled sera were commercially obtained independent of the processed EQAMs, resulting in different underlying matrices. They were explicitly not stabilized and spiked, and consisted of fresh pooled sera from male and female donors, aiming to achieve native concentrations of all three steroid hormones within the measuring ranges of the immunoassays used in the EQA survey and the accredited measuring range of the RMP. A bulk volume of up to 70 mL was reserved for stability testing and reference measurements in the calibration laboratory at INSTAND. Depending on the number of participants in the EQA survey, an additional 90–95 mL of each serum pool was immediately aliquoted into 0.5 mL portions. The bulk volume and the aliquots were delivered to INSTAND within 28 h after sampling. Sample preparation, shipment, and handling at INSTAND were performed at 2 °C–8 °C.

The stability of the three steroid hormones in the pooled native and unfrozen sera was assessed using a Roche cobas e411 chemiluminescence immunoassay analyzer. The samples were therefore stored under conditions reflecting those of the EQA surveys: at 2 °C–8 °C during shipment to INSTAND, followed by storage at room temperature during packaging and distribution to the participating laboratories. The first measurement was conducted immediately after sample receipt, i.e., one day after sampling. Subsequent measurements were performed daily throughout the EQA survey, and a final control measurement was carried out 1 week after sampling.

### 2.3 EQA procedure

The INSTAND EQA scheme for measuring testosterone, progesterone and 17β-estradiol is routinely conducted worldwide six times a year. Two 2 mL EQAMs at different concentrations are tested per survey (see Section 2.1). EQAMs are shipped to the participating laboratories at ambient temperature and must be analyzed within two and a half weeks. Participants should store the materials at 2 °C–8 °C until analysis. The quantitative results for testosterone, progesterone, and 17β-estradiol can be reported by the participating laboratories via the platform RV-Online (http://rv-online.instandev.de). In addition to the measurement results, participants should inform INSTAND as to which device, method and reagent they used.

In the EQA evaluation of all three steroid hormones, laboratories are allowed a maximum deviation of ±35% to the RMV to receive certification as stipulated by the guideline of the German Medical Association on quality assurance in medical laboratory examinations (Rili-BÄK) (Bundesärztekammer, 2023).

For three of the surveys in 2020 and 2021, the participating laboratories in Germany also received pooled native EQAMs alongside the two processed EQAMs at ambient temperature. A pilot survey was conducted in 01/2020 (month/year) in which one pooled native EQAM was dispatched for the measurement of progesterone. In 10/2020 and 10/2021, two additional pooled native EQAMs were shipped for the measurement of testosterone, progesterone and 17β-estradiol. In the three comparative EQA surveys, none of the native or processed EQAMs were used more than once. As the pooled native sera did not receive any stabilizing additives, shipment to the participating laboratories took place within 50 h after sample donation. Participants could voluntarily measure the additional native EQAMs, which was to be done within the following 3 days. The quantitative results for testosterone, progesterone, and 17β-estradiol as well as the device, method and assay used could be entered on the RV-Online platform.

The stability of the measurement of testosterone, progesterone and 17β-estradiol in the pooled native sera was monitored throughout the EQA survey (see Section 2.2).

### 2.4 Reference measurement procedures

RMPs are internationally recognized analytical methods of the highest metrological order. This makes the RMV ideally qualified as a target value for the evaluation of laboratory performance in external quality control. For all EQAMs used in this study, the RMVs for testosterone, progesterone and 17β-estradiol were determined by the INSTAND calibration laboratory, which is accredited according to EN-ISO/IEC (2018), EN-ISO/IEC (2019).

As established RMP for the three steroid hormones, isotope dilution gas chromatography/mass spectrometry (GC-ID/MS) was used. Metrological traceability was established using primary reference standards (testosterone NMIJ CRM 6002-a, progesterone NMIJ CRM 6003-a, 17β-estradiol NMIJ CRM 6004-a). Briefly, for the testosterone value assignments, samples were spiked gravimetrically with ^13^C_2_-testosterone as the internal standard and equilibrated, then precipitated with aqueous KOH, centrifuged and the supernatant was extracted into dichloromethane. Derivatization was performed with cyclohexane-heptafluorobutyric acid (HFBA) and subsequent partitioning into the cyclohexane phase.

GC-MS measurements were done at the mass-to-charge ratios of *m/z* 680 and *m/z* 682 (Thienpont et al., 1994). For progesterone measurements, samples were spiked gravimetrically with ^13^C_2_-progesterone as the internal standard and equilibrated, then extracted into n-hexane, followed by centrifugation and evaporation of the supernatant to dryness. Derivatization was performed with HFBA in cyclohexane. GC-MS measurements were done at *m/z* 510 and *m/z* 512. For the target value assignments for 17β-estradiol, the samples were spiked gravimetrically with ^13^C_2_-estradiol as the internal standard, equilibrated, then extracted into dichloromethane, followed by a clean-up step with Sephadex LH-20. Derivatization was performed with cyclohexane/acetone/HFBA. The GC-MS measurements were done at *m/z* 664 and *m/z* 666 (Siekmann, 1984). Six measurements were performed for each target value (two measurements per day over three consecutive days). Measurement uncertainty was assigned to each target value on the basis of a measurement uncertainty budget.

### 2.5 Data analysis and statistics

To assess the suitability of the six processed EQAMs for the measurement of testosterone, progesterone and 17β-estradiol, the corresponding EQA results were compared with those obtained from five to six native EQAMs. Since the suitability of a control material must be specifically considered for each MP, the EQA data for all subsequent analyses were divided into reagent manufacturer collectives. This study only takes into account those manufacturers for which at least three reported results per sample type and survey were available, i.e., Abbott (AB) with ARCHITECT or Alinity analytical systems, VIDAS from bioMérieux (AX), Access from Beckman (BE), Siemens, and Elecsys from Roche (RO). The results of the Siemens collective showed discrepancies across its sub-collectives, which were therefore analyzed separately. However, only the ADVIA Centaur (BG) and IMMULITE (DG) collectives, subordinate to Siemens, had at least three results per sample type and survey and were thus included in the manufacturer-specific analyses. For testosterone, the AIA system from Tosoh Bioscience (TH) was additionally considered, as the number of reported results for this collective has increased in recent years. However, voluntary measurements of the native samples by participants in the TH collective were largely not carried out in 10/2021. Consequently, comparative analyses of the results for the two sample types could only be performed for the survey in 10/2020.

Details on the assays and devices used by the participating laboratories are provided in the raw data (Supplementary Table S1).

The EQA results were transformed using the natural logarithm (ln), as the data were primarily positively skewed. Differences between the ln-transformed EQA results and the corresponding ln-transformed RMVs were calculated and are hereafter referred to as “ln bias.” Normalizing the EQA data to the RMVs in this way allowed us to summarize them cross-batch for each sample type and collective. The Grubbs test (α = 0.01) was used to identify outliers in the ln bias for each pairing of analyte, manufacturer collective, survey, and sample type. As a result, nine values for testosterone, seven values for progesterone and eleven values for 17β-estradiol were excluded from further analyses (see Supplementary Table S1).

**FIGURE 1:**
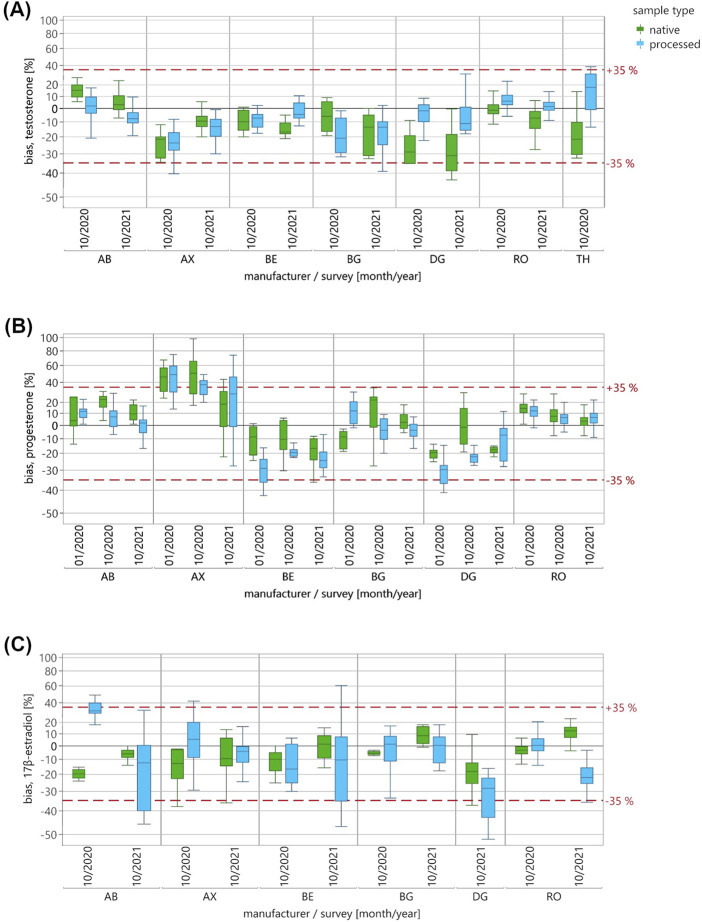
The distribution of the ln-transformed EQA results, normalized to the reference measurement value, for the measurement of **(A)** testosterone, **(B)** progesterone and **(C)** 17β-estradiol in pooled native (green) and processed (blue) EQAMs. Data are shown for each manufacturer collective and EQA survey using a percentage-scaled y-axis. A pilot survey was performed in 01/2020 with one pooled native EQAM and two processed EQAMs for the measurement of progesterone. In two further EQA surveys in 10/2020 and 10/2021, two pooled native and processed EQAMs were distributed each time for the measurement of the three steroid hormones. The whiskers of the boxes were defined to stretch from the 1st quartile −1.5 × (interquartile range) to the 3rd quartile +1.5 × (interquartile range). The red dashed line indicates the EQA evaluation criterion of +/−35% to the reference measurement value.

For each analyte, distributions of the sample type-specific ln bias to the RMV are shown as box plot diagrams for the individual manufacturer collectives and EQA surveys (Figure 1). The whiskers of the boxes were defined to stretch from the 1st quartile −1.5 × interquartile range to the 3rd quartile +1.5 × interquartile range. Statistical information on the ln bias distribution is provided in Supplementary Table S2.

In order to make the ln-transformed bias easier to interpret, the y-axis is scaled in percentages. The relationship is as follows:

$$bias\;\left[\%\right]=\left({e\;}^{\ln\;bias}-1\right)\;x\;100$$

The EQA evaluation criterion of ±35% to the RMVs, applicable to all three steroid hormones, is indicated by a red dashed line.

**FIGURE 2:**
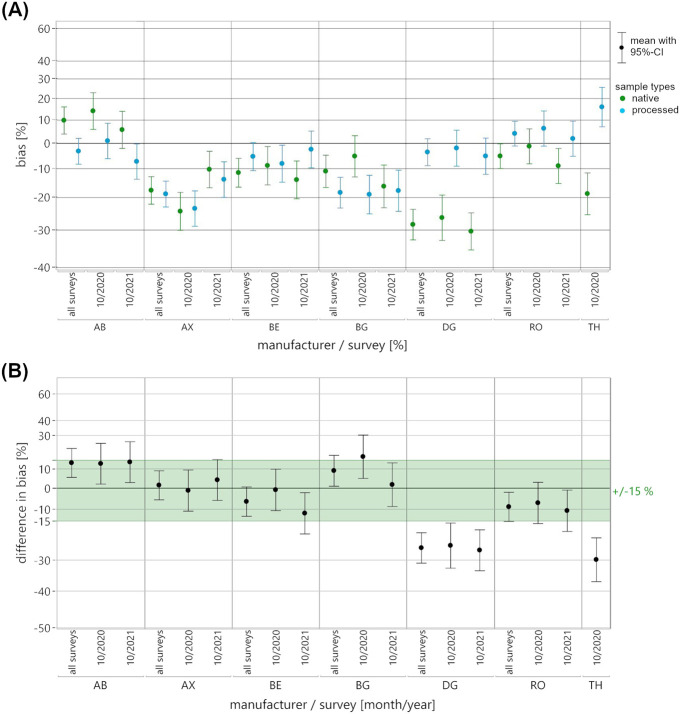
Results of the mixed-effects model of the ln-transformed EQA data for the measurement of testosterone in pooled native (green) and processed (blue) EQAMs. Data are shown for each manufacturer collective and EQA survey using a percentage-scaled y-axis. **(A)** The mean ln bias with a 95%-confidence interval (CI) is shown for both sample types, for each manufacturer collective and for both surveys (10/2020, 10/2021) as well as across the surveys (all surveys). **(B)** The difference in mean ln bias of the two sample types, bias of native minus bias of processed with 95%-CI, is shown for each manufacturer collective and both surveys as well as across the surveys (all surveys). For an initial assessment of the mean differences in bias, a commutability criterion was defined in consideration of clinical guidelines; this is shown as the light green area.

**FIGURE 3:**
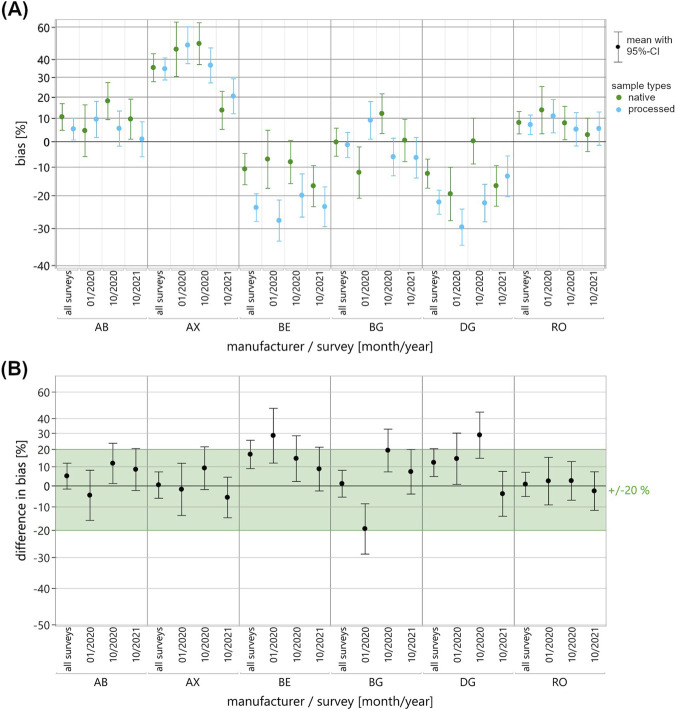
Results of the mixed-effects model of the ln-transformed EQA data for the measurement of progesterone in pooled native (green) and processed (blue) EQAMs. Data are shown for each manufacturer collective and EQA survey using a percentage-scaled y-axis. **(A)** The mean ln bias with a 95%-confidence interval (CI) is shown for both sample types, for each manufacturer collective and each survey (01/2020, 10/2020, 10/2021) as well as across the surveys (all surveys). **(B)** The difference in mean ln bias of the two sample types, bias of native minus bias of processed with 95%-CI, is shown for each manufacturer collective and each survey as well as across the surveys (all surveys). For an initial assessment of the mean differences in bias, a commutability criterion was defined in consideration of clinical guidelines; this is shown as the light green area.

**FIGURE 4:**
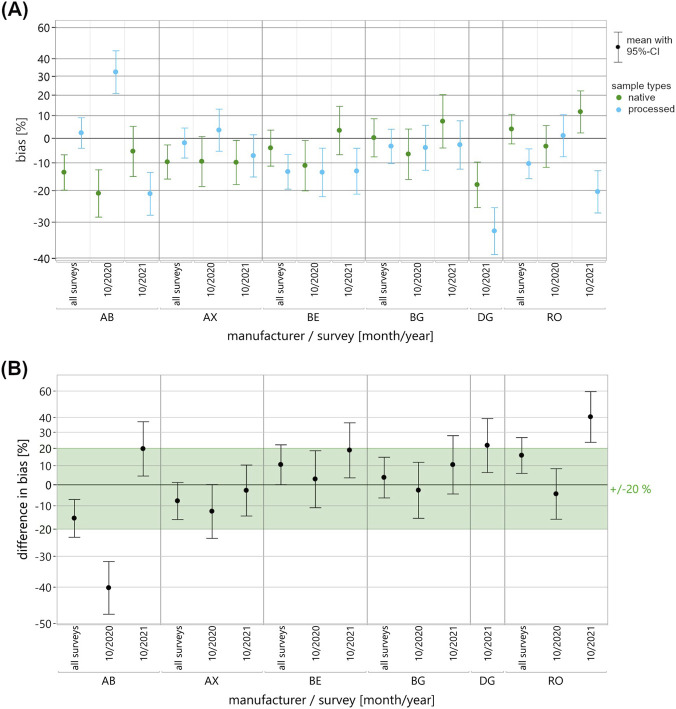
Results of the mixed-effects model of the ln-transformed EQA data for the measurement of 17β-estradiol in pooled native (green) and processed (blue) EQAMs. Data are shown for each manufacturer collective and EQA survey using a percentage-scaled y-axis. **(A)** The mean ln bias with a 95%-confidence interval (CI) is shown for both sample types, for each manufacturer collective and for both surveys (10/2020, 10/2021) as well as across the surveys (all surveys). **(B)** The difference in mean ln bias of the two sample types, bias of native minus bias of processed with 95%-CI, is shown for each manufacturer collective and both surveys as well as across the surveys (all surveys). For an initial assessment of the mean differences in bias, a commutability criterion was defined in consideration of clinical guidelines; this is shown as the light green area.

A mixed-effects model was employed to compare the EQA data of pooled native and processed EQAMs used in measuring testosterone, progesterone and 17β-estradiol. Fixed effects included “sample type” (native, processed), “manufacturer collective” (where applicable: AB, AX, BE, BG, DG, RO, TH), “survey” (where applicable: 01/2020, 10/2020, 10/2021), as well as all pairwise and three-way interactions. Random effects comprised “batch identification number” nested within the “sample type,” and “participant identification number” nested within the “manufacturer collective.” In response, first the mean ln bias to the RMV and then the mean differences in ln bias between the sample types were estimated, each with 95%-confidence intervals (CI). The results of the mixed-effects model were plotted for each manufacturer collective, as well as separately for each survey appointment and across the two or three appointments (Figures 2–4). Once again, the y-axis is scaled in percentages for easier interpretation of the illustrated data. For each pairing of analyte, manufacturer collective and survey, the mean bias observed for the two sample types was displayed directly next to each other (Figures 2A–4A).

The main results, i.e., the differences between the native and processed samples, are shown in the difference in bias plots, with commutability criteria for an initial assessment of the differences shown in light green (Figures 2B–4B). In this study, we set the acceptance criteria at ±15% for testosterone measurement and ±20% for progesterone and 17β-estradiol measurement, based on clinically relevant thresholds and specifications set out in endocrinology guidelines (Penzias et al., 2021; Bhasin et al., 2018; Elhassan et al., 2025; Mulhall et al., 2018). It is particularly important to take the endocrinological context (e.g., age, gender, or circadian rhythm) into consideration for the accurate diagnostic interpretation of sex hormone levels, which are subject to pronounced biological variability (Fraser, 2001; Ricós et al., 2019).

**FIGURE 5:**
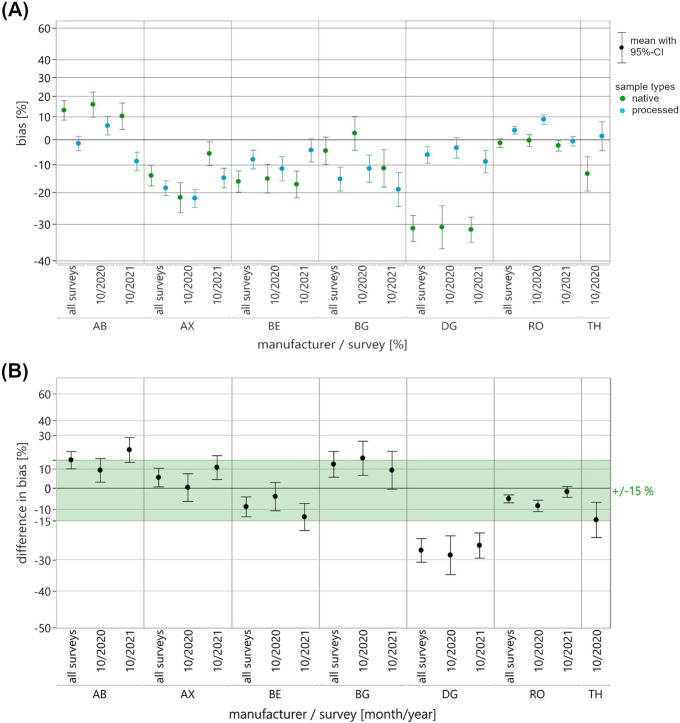
Results of the mixed-effects model of the EQA data for the measurement of testosterone in pooled native (green) and processed (blue) EQAMs with concentrations of 10–25 nmol/L. Data are shown for each manufacturer collective and EQA survey using a percentage-scaled y-axis. **(A)** The mean ln bias with a 95%-confidence interval (CI) is shown for both sample types, for each manufacturer collective and for both surveys (10/2020, 10/2021) as well as across the surveys (all surveys). **(B)** The difference in mean ln bias of the two sample types, bias of native minus bias of processed, with a 95%-CI is shown for each manufacturer collective and both surveys as well as across the surveys (all surveys). For an initial assessment of the mean differences in bias, a commutability criterion was defined in consideration of clinical guidelines; this is shown as the light green area.

MP-specific bias may depend on the analyte concentration, particularly for immunoassays with concentration-dependent selectivity. Inclusion of analyte concentration as a fixed effect in the mixed-effects model was not considered appropriate due to the limited number of discrete concentration levels, which would have provided limited statistical robustness. However, since remarkable concentration-dependent effects were observed in a previous study for the testosterone/TH pairing (Vierbaum et al., 2024b), a mixed-effects model analysis was restricted to EQAMs in the range of 10–25 nmol/L testosterone, in order to achieve better comparability of sample concentrations and to examine the effects on the model outcomes (Figure 5). Since this restriction further reduces the already low number of EQAMs, minor effects on the model outcomes are expected and should be interpreted with caution.

All statistical analyses were performed by JMP 18.2.2 from SAS Institute (Cary, North Carolina, USA).

### 2.6 Generation of images

The overlay images were generated using the Gnu image manipulator software 2.10.38.

## 3 Results

As the measurement of native EQAMs was voluntary for participating laboratories, fewer measurement results were submitted in some cases than for the processed EQAMs. Overall, this corresponded to a participation rate of approximately 65%, with non-participation primarily due to organizational reasons rather than systematic bias in laboratory performance. In the first EQA survey in 01/2020, the voluntary measurement of progesterone in the pooled native EQAMs led to 4 to 8 reported results for each reagent manufacturer collective, except the RO collective which had 30 reported measurement results. In the two subsequent EQA surveys for the measurement of testosterone, progesterone and 17β-estradiol, which were conducted in 10/2020 and 10/2021, the number of results obtained for each manufacturer collective and survey varied from 6 to 22, but was between 76 and 96 for the RO collective (Supplementary Table S1).

The distribution of the sample type-specific EQA measurement results for the three analytes is shown for each manufacturer collective and EQA survey (Figure 1). The mean bias to the RMVs varied across manufacturer collectives, showing both positive and negative deviations, and in some cases values exceeded the EQA acceptance criterion of ±35%.

For testosterone, negative deviations from the RMV were observed for the AX, BG and DG collectives, with individual results below the EQA criterion of −35% (Figure 1A). For the DG collective, strong deviations of up to −31% in the median bias were observed only for the pooled native EQAMs, whereas the processed samples showed a maximum deviation of −11%. The TH collective also displayed discrepant results between sample types, with upward deviations for processed EQAMs and downward deviations for pooled native EQAMs.

For progesterone, the AX collective showed values higher than the RMV, with several results exceeding the EQA criterion for both pooled native and processed EQAMs (Figure 1B). The BE and DG collectives also exceeded the lower limit of the criterion, but almost exclusively for processed EQAM. The median bias of the other collectives deviated by less than 23% from the RMVs in all surveys.

For 17β-estradiol, the majority of the collectives showed median deviations to the RMVs of less than ±13% (Figure 1C). Notable exceptions with high bias were the AB collective in 10/2020 and the DG and RO collectives in 10/2021, all of which showed higher bias for processed than for pooled native EQAMs. For all collectives, both sample types demonstrated both positive and negative deviations from the RMVs. Some individual EQA results did not meet the EQA acceptance criterion of ±35%.

For the three analytes, individual manufacturer collectives showed remarkably consistent mean bias, while others demonstrated considerable variability across the two or three surveys (Figures 2A–4A). A visual comparison of mean bias across the pairings of analyte and reagent manufacturer indicated discrepancies between the two sample types, occurring either sporadically in individual surveys (e.g., 17β-estradiol; RO) or systematically across all surveys (e.g., testosterone; AB and DG). For some manufacturer collectives, higher bias was observed in one survey for pooled native EQAMs and in another survey for processed EQAMs (e.g., progesterone; AB, AX, BG, or DG).

Considerable discrepancies in mean bias between the pooled native and processed EQAMs in individual surveys were reflected by substantial differences in bias (Figures 2B–4B). Changes in the relationship of mean bias between sample types across surveys (Figures 2A–4A) is accompanied by high variability in the calculated differences (Figures 2B–4B). A straightforward visual classification of the observed differences in bias between pooled native and processed EQAMs indicated that most of the mean differences were within the analyte-specific commutability criterion (Figures 2B–4B). However, some exceptions could be observed.

With regard to the measurement of testosterone, the largest difference in bias was found to be −29.9% for the TH reagent, with data only available for a single survey (10/2020). Similar substantial differences of approximately −25.7% were noted for the DG reagent in both the 10/2020 and 10/2021 surveys (Figure 2B). This is due to the previously mentioned constant relationship in mean bias between pooled native and processed EQAMs (Figure 2A).

For progesterone, large mean differences in bias between the sample types were observed in individual surveys for the collectives BE (28.6%), BG (−19.3% and 19.4%), and DG (28.9%) (Figure 3B). Because there was a higher mean bias for native EQAMs in some surveys and for the processed EQAMs in others (Figure 3A), there was a high variability in the differences in bias with both positive and negative values in the different surveys (Figure 3B). For progesterone, the largest variability in mean differences in bias was demonstrated by the BG collective, ranging from −19.3% to 19.4%, followed by the DG collective with −3.8%–28.9%. Both collectives showed considerable variability in mean bias for each sample type (Figure 3A). In the case of the BG collective, a slightly higher variation was observed for native than for processed EQAMs, ranging from −12.0% to 12.1% for the native EQAMs. In the case of the DG collective, mean bias varied similar for the native and processed EQAMs, with a tendency toward slightly more negative bias for the processed EQAMs down to −29.6%. The AX and BE collectives also showed similar variability in mean bias, but the relationship between native and processed EQAMs changed less, resulting in smaller differences.

For 17β-estradiol, two conspicuous differences of −40.3% for the AB collective and 40.5% for the RO collective were found for an individual survey (Figure 4B). For both collectives, a high variability in the differences correlated with high variabilities in the mean bias (Figure 4A). In both cases, the variability in mean bias was found to be greater for processed EQAMs (AB: −21.1%–32.3%; RO: −20.4%–1.0%) than for the corresponding pooled native EQAMs.

When mixed-effects model analyses were conducted that only considered EQAMs with mid-range levels of testosterone of 10–25 nmol/L, most manufacturers showed only slight increases or decreases in the differences in bias between the two sample types (Figure 5) compared to the outcomes of the initial analyses (Figure 2). Slight changes were expected due to the reduced number of EQA batches involved in the analyses. Compared to the initial analyses, the changes in the difference in bias remained around 5% in most cases. In the initial mixed-effects model analyses of the testosterone measurements, taking all EQAMs into account, the DG and TH collectives showed strikingly high mean differences in bias between the two sample types (Figure 2). The difference initially observed for the TH collective was significantly reduced from −29.9% to −14.6% when only EQAMs with testosterone levels of 10–25 nmol/L were considered (Figure 5). In contrast, there were no remarkable changes in the large differences observed in the mean bias for the DG collective when only EQAMs with testosterone levels of 10–25 nmol/L were compared.

## 4 Discussion

The present study investigated whether comparative EQA analyses using a limited number of native EQAMs alongside routinely distributed processed EQAMs can provide meaningful information on the suitability of the processed EQAMs used across several MPs. In order to justify the universal, cross-MP use of processed reference materials–such as for the metrological traceability of MP-specific values or external quality control–it is essential to minimize any systematic deviation of MP-specific measurement results from those obtained using patient samples (Jennings et al., 2019; Miller et al., 2018; Nilsson et al., 2018). However, when EQA evaluations reveal measurement differences between MP collectives or when compared to the RMV, it is not trivial to determine the extent to which this is affected by the materials or the MPs. Influences resulting from the processed nature of the control materials are particularly critical if the accuracy of the laboratory results is evaluated relative to the RMV as the assigned target value (Ceriotti, 2014; Franzini and Ceriotti, 1998; Miller, 2003; Miller et al., 2011a).

International consortia such as The International Federation of Clinical Chemistry and Laboratory Medicine (IFCC) Working Group on Commutability in Metrological Traceability describe comprehensive study designs for assessing the commutability of processed control materials (Bland and Altman, 1986; Miller et al., 2018; Nilsson et al., 2018). In practice, such studies are limited by restricted access to several market-leading medical devices and to patient materials, especially in pathological concentration ranges (Vierbaum et al., 2024a). Consequently, statements about the commutability of control materials are usually only valid within the context of very specific study designs, such as the selected MPs or the concentration ranges achieved for the patient samples. Particularly for control materials manufactured with a relatively high batch frequency, such as EQA batches, comprehensive commutability studies can only be carried out sporadically for selected analytes, MPs, and EQAMs. Since the recommended approaches for assessing commutability pose major challenges for EQA providers in terms of practical implementation and are very resource-intensive, this study investigates the extent to which a comparison of EQA data of occasionally distributed native EQAMs to the data of regularly distributed processed EQAMs can provide information about the comparability of the different sample types. This approach utilizes the infrastructure and routine processes of EQA providers but also entails challenges that go beyond routine tasks and resource demands that should not be understated. As commutability studies require that patient samples be measured in a fresh, non-stabilized state, with samples in this study even distributed unfrozen, EQA management is highly time sensitive. This applies from the moment the sample is donated, through to aliquoting, logistical handling, and measurement in the participating laboratories. Furthermore, short-term donation cancellations must be anticipated in addition to the already low number of native samples–neither of which occurred in the surveys conducted for this study. Of course, comparative analyses of the EQA data for both sample types become more representative and meaningful as the number of native EQAMs increases. Using a limited number of EQAMs carries the risk of missing sample-specific effects, e.g., caused by medication or specifically by hormone supplementation (Cirrincione et al., 2022; Jeffery et al., 2014). However, a major strength of the comparative EQA study design is that data are collected for the actual users of the processed EQAMs and, ultimately, for all the market-relevant MPs. In conventional commutability assessments, one significant limitation is that usually only selected MPs can be considered (Miller et al., 2026; Vierbaum et al., 2024a). Sample type comparisons in comparative EQA studies can be based on reliable average bias obtained for the MP collectives, whereas conventional commutability studies typically rely on measurements from single laboratories for each MP. Naturally, the representativeness of an average increases with the size of the individual MP collectives; however, these sizes are specific to each EQA program and can only be influenced to a limited extent.

With respect to the measurement of the three steroid hormones in processed EQAMs, previous longitudinal analyses of EQA data have revealed, among other things, remarkable deviations from the RMVs for individual reagent collectives, with individual values of certain collectives repeatedly exceeding the EQA evaluation criterion (Vierbaum et al., 2024b). Hence, this EQA-based approach using comparative native EQAMs aimed to distinguish potential non-commutability bias from trueness bias due to intrinsic MP performances, based on estimated mean bias to the RMV for native and processed materials and differences in bias between sample types. A similar reagent-specific mean bias for processed and native EQAMs would suggest that observed measurement deviations are primarily attributable to trueness bias rather than non-commutability bias of the routinely distributed processed EQAMs. Thus, slight mean differences in bias observed between the two sample types would argue for patient-like materials.

To get to a quantitative assessment of the suitability of the processed EQAMs based on the mixed-effects model results, it is necessary to define a maximum acceptable difference between the sample types. While the definition of an appropriate commutability criterion is ultimately decisive in the evaluation of control materials, there is currently no standardized approach or consensus on this matter (Vierbaum et al., 2024a). Miller et al. recommend, with focus on the assessment of certified reference materials (CRMs), defining the criterion for assessing commutability as a fraction of the maximum permissible combined standard uncertainty for clinical sample results (Miller et al., 2023). This reports a clinically and statistically adequate definition of a criterion considering measurement uncertainties and error budget; however, they point out that practical implementation may well be not realizable due to the extensive study designs required. Accordingly, alternative statistically defined and often less stringent criteria are expedient for practical application, particularly for the assessment of EQAMs. Sandberg et al. present a recommendation for the definition of commutability criteria in EQA (Sandberg et al., 2023). But, given the limited statistical power to characterize the variability in bias to the RMV of native EQAMs in this study, it is reasonable to initially apply a rather strict patient-centered criterion defined in consideration with clinical guidelines and reference ranges (Penzias et al., 2021; Bhasin et al., 2018; Elhassan et al., 2025; Mulhall et al., 2018) (Figures 2–5B, see light green range).

In one analyte/manufacturer pairing, testosterone/DG, persistent differences in bias were observed beyond the commutability criterion across all surveys (Figure 2). For other pairings, differences exceeding the respective criteria were only observed for individual EQA surveys. This was the case for testosterone measurement using the BG reagent, with a slight deviation from the acceptance criterion in one survey (Figure 2). More pronounced deviations from the criterion were observed with regard to progesterone measurement in individual surveys for the BE, BG, and DG collectives (Figure 3) and for 17β-estradiol measurement for the AB and RO collectives (Figure 4). For two pairings, data for only one survey were available: testosterone measurement with the TH reagent and 17β-estradiol measurement with the DG reagent. In both cases, differences beyond the commutability criterion were observed, but additional surveys using native EQAMs could help to verify the reliability of these findings.

In nine out of a total of nineteen pairings, the difference in bias exceeded the acceptance criterion in at least one survey. Thus, the use of criteria defined in consideration of clinical guidelines enables an initial differentiation of analyte/manufacturer pairings with potentially clinically relevant and irrelevant differences in bias between processed and native EQAMs, at least for the investigated EQAMs considered.

When applying maximum allowable percentage deviations as commutability criteria, however, the influence of the analytical performance of the individual MPs has not yet been considered. When assessing the suitability of EQA control materials, it is advisable to distinguish between non-commutability bias and trueness bias. If, theoretically, many native samples were included, there would be a maximum observable bias range depending on the respective MP performance–the optimal MP-specific bias distribution achievable under ideal sample conditions. Using a limited number of native EQAMs - five or six in this study - results in random bias falling within this theoretical maximum bias range. Nevertheless, provided that the bias range observed in the native EQAMs exceeds that of processed EQAMs, non-commutability bias cannot be entirely ruled out but are modest relative to the variability in bias attributable to MP performance. An example of this situation is the measurement of progesterone with the BG reagent (Figure 3). For the measurement of progesterone with the DG reagent and testosterone with the BG reagent, variability in bias was observed to be higher for the native than for the processed EQAMs, however, there was a slight shift between the bias ranges of the two sample types. In the case of progesterone measurement with the DG reagent, the high variability in bias observed for the native EQAMs revealed issues related to the insufficient analytical performance of the MP. If mean bias estimates are based on small collectives in some pairings, it must be considered that laboratory-specific analytical inconsistencies may have an impact.

Conversely, if considerable variability in the bias is primarily observed for the processed EQAMs or exceeds the scatter range of native EQAMs, this may indicate issues with sample suitability that would need to be further investigated. In the case of 17β-estradiol measurement using the AB and the RO reagents, the variability in bias was found to be high for both sample types, but the scatter for the processed EQAMs was substantially greater than for the pooled native EQAMs. The conspicuously high bias for processed EQAMs is probably due to modifications in the matrix or sample additives that interfere with 17β-estradiol measurement for the respective reagents. But it is also possible that the lower scatter in the bias observed for the limited number of native EQAMs appeared by chance and would increase if additional native EQAMs were included in future EQA surveys. The analysis of the retrospective EQA results for steroid hormones (Vierbaum et al., 2024b) shows that the MP-specific 17β-estradiol results deviated from the RMV depending on the concentration, at least for the processed EQAMs. This would also be conceivable for the native EQAMs.

Fairly reliable conclusions can be drawn from the results of comparison studies when consistent bias is observed across surveys for the individual sample types, suggesting great MP performance and reliable measurements. When combined with minor differences in bias, it suggests that no interference arises from the processed material, e.g., progesterone measurement with the RO reagent (Figure 3).

If (relatively) consistent bias is accompanied by differences in bias exceeding the commutability criterion, interferences are likely to be caused by the processed nature of the EQAMs like that which is observed for the testosterone measurement with the DG reagent (Figure 2) or progesterone measurement with the BE reagent (Figure 3). In such cases, the use of processed materials in EQA can lead to biased measurement results compared to actual analytical performance in routine diagnostics, making EQA evaluations based on the RMV invalid. In the case of testosterone measurement with the DG reagent, an inaccurate measurement would go unnoticed in an EQA evaluation, as the processed EQAMs showed a bias of only about −3.6% compared to −28.4% for the native EQAMs. The processed EQAMs examined in this study are therefore unsuitable for assessing based on EQA results the accuracy of the DG collective and can only serve to evaluate collective-specific precision. In the case of progesterone measurement using the BE reagent, processed EQAMs tend to deviate more from the RMV than the native EQAMs. The use of these processed EQAMs in EQA could potentially put the laboratories within this collective at a disadvantage, if evaluated against the RMV. Corrective measures based on the EQA results would be inappropriate. However, among the three surveys conducted, only one showed a bias exceeding the clinically relevant threshold of ±20%. To confirm this trend and gain deeper insight into the variability of native materials, additional EQA surveys using native control materials would be beneficial.

Another important factor that would influence the commutability assessment of EQAMs would be the potential concentration dependencies of MP-specific bias. The accurate quantification of steroid hormones using immunoassays can be challenging due to a susceptibility to cross-reactions with other steroids, particularly for samples with low steroid hormone concentrations (Cao et al., 2019; Kanakis et al., 2019; La'ulu et al., 2018; Middle, 2007; Vierbaum et al., 2024b). However, in the present study, analyte concentration was not included as a covariate in the model analysis due to the limited number of discrete EQAM levels, which restricts statistical robustness. Previous longitudinal EQA analyses demonstrated pronounced concentration-dependent bias for testosterone using the TH reagent, with underestimation at low and overestimation at higher concentrations (Vierbaum et al., 2024b). To illustrate the potential impact of this limitation, testosterone was used as an example, and analyses were repeated within a restricted concentration range (10–25 nmol/L). While only minor changes were observed for most collectives (e.g., DG), a substantial reduction in bias differences was seen for the TH reagent (−14.6% instead of −29.9%), indicating that concentration-dependent effects influenced the initial results. These findings highlight that, in the absence of explicit modeling of concentration, MP-specific concentration-dependent bias should be investigated separately.

All in all, after considering both the commutability criterion defined in consideration with clinical guidelines and a statistically related criterion - using the bias range of native EQAMs as the reference range for the bias observed for processed EQAMs - processed EQAMs were found to be very likely non-commutable for two analyte/manufacturer pairings: testosterone/DG and progesterone/BE. The latter exhibited commutability issues in just one survey. For the determination of 17β-estradiol, inconsistent, strongly scattered bias was observed with the use of certain reagents, making it difficult to assess commutability. Hence, commutability remained inconclusive in individual surveys for four further analyte/MP pairings, requiring further data from a larger number of native EQAMs. But despite the mentioned exceptions, processed EQAMs for measuring testosterone, progesterone and 17β-estradiol were found to be suitable for EQA purposes.

In general, the inclusion of additional native samples in future EQA surveys can improve the reliability of the comparative analyses and the interpretation of results. However, it must be considered that manufacturers could implement batch changes or modify assay procedures and calibrations between successive surveys as this would potentially affect the temporal stability of the measurement bias but not be due to unreliable MP performance. For example, INSTAND has received information about a corresponding test modification for the measurement of testosterone from a manufacturer (Vierbaum et al., 2024b). While the AX assay had repeatedly shown unsatisfactory passing rates in previous EQA surveys, a significant and lasting reduction in bias to the RMV below −15% in the median was reported for early 2021.

In this study, the AX collective showed minor mean differences in bias between the two sample types of −1.1% in 10/2020 and 4.2% in 10/2021 (Figure 2B). The comparative EQA survey in 10/2020 provided initial evidence that the observed bias was attributable to trueness bias (e.g., insufficient calibration or assay specificity) rather than non-commutability. While the mean bias for both sample types was approximately −24% in the 10/2020 survey, it was less than −14% in the 10/2021 survey, suggesting improved accuracy of the MP. This improvement may have resulted from recalibration efforts in terms of metrological traceability.

This example demonstrates the potential of EQA studies with comparative native materials, provided that there are consistently, and thus reliably, low differences in bias between the sample types. Based on the remarkably high bias for both sample types and observed differences in bias of less than 10% (Figure 3), corrective actions in the progesterone assay of AX would be recommended as well.

Overall, incorporating comparative native materials into the EQA on a sporadic basis–like every one to 2 years, depending on how critical the issue of EQAM commutativity is in each specific case - provides valuable insights into the accuracy of the individual MPs. In complement to insights into trueness bias, this approach allows pairings of analytes and reagent manufacturers to be identified that may be critical or less critical in terms of the commutability of processed EQAMs. In particular, reliable and consistent bias allows for the most robust conclusions with regard to the suitability of processed materials. Inconclusive findings indicate the need to include additional native EQAMs. If small collective sizes are involved, such as in this EQA scheme for the measurement of steroid hormones, a higher number of native EQAMs within a survey can improve the robustness of the data. To further strengthen the data basis when implementing the proposed approach in the future, introducing mandatory measurements of sporadic native EQAMs for participating laboratories within the EQA scheme should be contemplated, e.g., by designating the native EQAMs as commutable anchor samples for the purpose of accuracy control. To simplify the compact and time-sensitive EQA preparation process - from blood donation through sample preparation, aliquoting, and shipping to participating laboratories - it may be appropriate to temporarily freeze freshly prepared, native donor samples for use as native EQAM (Miller et al., 2008).

Based on processed EQAMs identified as commutable, the commutability of identically produced batches can be assumed in the first instance, due to their highly standardized manufacturing processes, with some caution regarding potential sample-specific effects. However, to recognize sample-specific effects of individual EQAM batches, MP-specific biases can be monitored longitudinally. In addition, MP developments and assay batch changes over time should be followed closely.

Pairings of analyte and MP, for which a lack of commutability remains likely based on the outcome of comparative EQA studies, can be specifically selected for more extensive commutability assessments. In particular, as the number of MPs involved in conventional commutability assessments is inherently constrained (Miller et al., 2026; Vierbaum et al., 2024a), strategic pre-selection of critical analyte/MP pairings further optimizes the use of limited experimental capacity. In case the commutability of processed EQAMs is insufficient or remains unknown, an evaluation of EQA results can only be appropriately made based on consensus values.

## 5 Conclusion

Accuracy-based EQA evaluations and evidence-based corrective actions for individual MPs are only appropriate when commutable EQAMs are used. Given the practical limitations and logistical constraints of comprehensive commutability studies, a pre-identification of potentially critical analyte/MP pairings in EQA studies with comparative native materials, followed by targeted clarifying commutability assessments is an efficient strategy for resource allocation. Nevertheless, even these more pragmatic approaches remain resource-intensive and are inherently restricted to specific questions and selected analytes.

## Data Availability

The original contributions presented in the study are included in the article/Supplementary Material, further inquiries can be directed to the corresponding author.
